# Filovirus VP24 Proteins Differentially Regulate RIG-I and MDA5-Dependent Type I and III Interferon Promoter Activation

**DOI:** 10.3389/fimmu.2021.694105

**Published:** 2022-01-05

**Authors:** Felix B. He, Hira Khan, Moona Huttunen, Pekka Kolehmainen, Krister Melén, Sari Maljanen, Mengmeng Qu, Miao Jiang, Laura Kakkola, Ilkka Julkunen

**Affiliations:** ^1^ Institute of Biomedicine/Virology, University of Turku, Turku, Finland; ^2^ Expert Microbiology Unit, Finnish Institute for Health and Welfare, Helsinki, Finland; ^3^ Research Center for Clinical & Translational Medicine, Fifth Medical Center for General Hospital of People’s Liberation Army (PLA), Beijing, China; ^4^ Turku University Hospital, Clinical Microbiology, Turku, Finland

**Keywords:** ebolavirus, filovirus, innate immunity, RIG-I pathway, MDA5 pathway, VP24, type I interferon, type III interferon

## Abstract

Filovirus family consists of highly pathogenic viruses that have caused fatal outbreaks especially in many African countries. Previously, research focus has been on Ebola, Sudan and Marburg viruses leaving other filoviruses less well studied. Filoviruses, in general, pose a significant global threat since they are highly virulent and potentially transmissible between humans causing sporadic infections and local or widespread epidemics. Filoviruses have the ability to downregulate innate immunity, and especially viral protein 24 (VP24), VP35 and VP40 have variably been shown to interfere with interferon (IFN) gene expression and signaling. Here we systematically analyzed the ability of VP24 proteins of nine filovirus family members to interfere with retinoic acid-inducible gene I (RIG-I) and melanoma differentiation-associated antigen 5 (MDA5) induced IFN-β and IFN-λ1 promoter activation. All VP24 proteins were localized both in the cell cytoplasm and nucleus in variable amounts. VP24 proteins of Zaire and Sudan ebolaviruses, Lloviu, Taï Forest, Reston, Marburg and Bundibugyo viruses (EBOV, SUDV, LLOV, TAFV, RESTV, MARV and BDBV, respectively) were found to inhibit both RIG-I and MDA5 stimulated IFN-β and IFN-λ1 promoter activation. The inhibition takes place downstream of interferon regulatory factor 3 phosphorylation suggesting the inhibition to occur in the nucleus. VP24 proteins of Mengla (MLAV) or Bombali viruses (BOMV) did not inhibit IFN-β or IFN-λ1 promoter activation. Six ebolavirus VP24s and Lloviu VP24 bound tightly, whereas MARV and MLAV VP24s bound weakly, to importin α5, the subtype that regulates the nuclear import of STAT complexes. MARV and MLAV VP24 binding to importin α5 was very weak. Our data provides new information on the innate immune inhibitory mechanisms of filovirus VP24 proteins, which may contribute to the pathogenesis of filovirus infections.

## Introduction

Filoviruses belong to the order of *Mononegavirales* and *Filoviridae* is one of the eleven families (1). Characteristics of filoviruses are filamentous virion structure, long genomes containing overlapping genes, transcriptional initiation and termination signals, and unique structural proteins without obvious structural and functional homologs with other mononegavirus species (2). Filoviruses are divided into six genera: *Ebolavirus, Marburgvirus, Cuevavirus, Dianlovirus, Striavirus* and *Thamnovirus*. Of these viruses *Zaire ebolavirus* (EBOV), *Sudan ebolavirus* (SUDV) and *Marburgvirus* (MARV) have caused severe outbreaks and the infection is characterized with a high mortality in humans (3). So far, the largest ebolavirus outbreak with a clinical syndrome named as Ebola virus disease (EVD), took place in West Africa in 2014–2015, resulting in more than 28 000 cases with 11 300 deaths (4). Among the twelve viruses assigned to the family of filoviruses, EBOV, SUDV and MARV are the most characterized ones mainly due to their high lethality in infection outbreaks (5).

Filoviruses consist of ssRNA genomes of 19 kilonucleotides and the genomes encode up to eight translation products derived from seven separate transcriptional units (6). The gene order of EBOV genome is nucleoprotein (NP), viral protein (VP) 35, VP40, glycoprotein/secreted glycoprotein (GP/sGP), VP30, VP24, and viral polymerase L. Out of these eight proteins, VP35 and VP24 have been shown to interfere with the activation of host innate immune responses. VP35 of both EBOV and MARV inhibit the interaction of viral RNA with retinoic acid-inducible gene I (RIG-I) by binding to and sequestering viral dsRNA leading to impaired activation of IRF3. EBOV VP35 has shown to interfere with the RIG-I ATPase activation by disrupting the interaction between protein kinase R (PKR) or PACT activator with RIG-I (7). VP35 can also inhibit the functions of IkappaB kinase-epsilon/TANK-binding kinase-1 (IKKϵ/TBK1) complex (8). VP24 (EBOV VP24) is a minor matrix protein, which is also able to inhibit IFN-induced antiviral responses. It binds to cellular importin α molecules, especially importin α5 and α6 and thus the nuclear import of phosphorylated STAT1 and STAT1-STAT2 dimers is prevented, leading to impaired Janus kinase–signal transducer and activator of transcription (Jak-STAT) signaling and antiviral responses (9). VP24 also efficiently inhibits IFN gene expression downstream of the RIG-I pathway and a recent study showed that this event likely takes place in the nucleus (10). Previous studies demonstrated that ebolavirus species VP24 proteins, but not that of MARV, can antagonize IFN gene expression (11, 12). A common feature for all ebolavirus family VP24s and MARV VP40 is that they efficiently interfere with IFN signaling and IFN-induced gene expression (12, 13).

Invading and replicating viruses are being recognized by the host cell via pattern recognition receptors (PRRs). PRRs recognize pathogens via pathogen-associated molecular patterns (PAMPs) and activate host innate immune responses. The production of antiviral proteins and initiation of adaptive immune response is mediated through different cellular signaling pathways that activate the production of type I (IFN-α/β) and type III interferons (IFN-λ). Infection of cells with an RNA virus activates different PRRs, such as Toll-like receptors (TLRs), RIG-I-like receptors (RLRs) and nucleotide-binding oligomerization domain-containing (NOD)-like receptors (NLRs). RIG-I and melanoma differentiation-associated antigen 5 (MDA5) are activated by viral ss/dsRNA molecules leading to activation and nuclear translocation of IRF3 and IRF7, NF-kB and MAPK TFs (14–16). Binding of secreted type I and III IFNs to their specific receptors results in the activation of Jak–STAT signaling pathway which ultimately leads to the enhanced expression of IFN-stimulated genes (17) such as antiviral proteins MxA, MxB, Viperin, IFITMs, OAS and PKR, just to mention some of them.

In the present study we focused on potential functional differences of filovirus VP24 proteins. We found that EBOV, SUDV Bundibugyo (BDBV), Reston (RESTV), Taï Forest (TAFV), Lloviu virus (LLOV) and MARV VP24s, but not Bombali (BOMV) and Mengla virus (MLAV), efficiently inhibited both RIG-I and MDA5-dependent type I and III interferon promoter activation. IRF3 phosphorylation was not inhibited by selected VP24s and EBOV VP24 did not inhibit the nuclear import of activated IRF3. The data obtained provides new information on the ability of filoviruses to delay or interfere with the activation of innate immune responses, a phenomenon important in understanding virus pathogenesis and disease progression as well as in identifying potential new drug targets.

## Materials and Methods

### Cell Culture, Transfections and Cell Stimulations

Human embryonic kidney 293 (HEK293) and human hepatoma Huh7 cells were maintained in Dulbecco’s modified Eagle’s medium (D-MEM) supplemented with HEPES (MP Biomedicals).

Immortalized RIG-I^+/+^/MDA5^+/+^(wild type), RIG-I^-/-^/MDA5^+/+^, RIG-I^+/+^/MDA5^-/-^ and RIG-I^-/-^/MDA5^-/-^ mouse embryonic fibroblast (MEF) cell lines were maintained as described previously (18). Cell media was supplemented with 10% heat inactivated fetal bovine serum (FBS, Biowest), penicillin/streptomycin and L-glutamine. Plasmids were transfected into HEK293 and Huh7 cells with TransIT-LT1 Reagent according to manufacturer’s instructions (Mirus Bio LCC, Madison). PolyI:C stimulation was carried out by first transfecting plasmids with TransIT-LT1 reagent followed by transfection with stimulatory low molecular weight polyI:C (10μg/well in 12-well plates; LMW polyI:C; InvivoGen) with Lipofectamine2000 (Invitrogen). Ebolavirus ssRNA mimic was transfected into MEF cells using Lipofectamine3000 transfection reagent (Invitrogen) (19).

*Spodoptera frugiperda* (Sf9) (ATCC: CRL-1711) cells were used for baculovirus expression and they were maintained in TNM-FH medium (Sigma-Aldrich Co.) supplemented with 0.6 µg/ml penicillin, 60 µg/ml streptomycin, 2.5 µg/ml amphotericin B and 10% fetal calf serum (Sigma-Aldrich Co.) or in EX-C420 Serum-Free Medium (Sigma-Aldrich Co.) as recommend by the manufacturer.

### Plasmids and RNA Constructs

Sequences for the VP24 of EBOV (KM233113), BOMV (MF319185), BDBV (KC545394), RESTV (KY798006), SUDV (KC545389), TAFV (KU182910), LLOV (NC016144.1), MARV (NC001608.3) and MLAV (KX371887) were retrieved from GenBank (https://www.ncbi.nlm.nih.gov/) and the VP24 genes were synthesized by Geneart (Thermo Fisher Scientific). Due to low protein production encoded by authentic BDBV and MARV VP24 genes codon optimized versions of the respective genes were obtained. The genes were cloned into an expression plasmid pEBB-HA-N, (a kind gift from Professors Kalle Saksela and David Baltimore), which creates a fusion protein with an N-terminal HA-tag. Nuclear localization signal (NLS) (or importin α interaction site) mutated EBOV VP24 expression plasmid has been described previously (10). Luciferase reporter plasmids pIFN-β-Luc and pIFN-λ1-Luc containing the promotor areas for the interferon genes in front of a firefly luciferase were used (20). Renilla luciferase gene under Rous sarcoma virus promoter (RSV-Renilla), as well as the expression plasmids for wtRIG-I, constitutively active form of RIG-I (ΔRIG-I) (21), MDA5 (22), wtIRF3 (23) and hepatitis C virus nonstructural protein 3/4A (HCV NS3/4A) (24) have been described previously.

A minigenome of EBOV (EBOV_negssDNA_GFP, 1940 bp) was designed as a reverse complement to the virus genome with a chimeric eGFP gene between 5’UTR trailer and 3’UTR leader regions of the genome of EBOV (KM233113) (**Supplementary Figure 1**). The fragment was synthesized and cloned into pMKRQ vector backbone (Geneart, Thermo Fisher Scientific). The plasmid was used as the template for PCR to produce EBOV dsDNA with a T7 promoter. The oligonucleotides used in the PCR reaction were 5’-CGCGTAATACGACTCACTATAGGGACACACAAAAAAGAAGAA-3’ (sense primer, T7 promoter underlined) and 5’-CGGACACACAAAAAGAAAGAAGAAT-3’ (antisense primer). EBOV dsDNA was used as the template to produce an ssRNA with the authentic EBOV genome ends: virus 3’UTR leader followed by eGFP gene and virus 5’UTR trailer. The ssRNA-product was extracted and analyzed as previously described (15, 19). *In vitro* produced ssRNA was further purified and desalted with NAP5 column (GE Healthcare).

The N-terminal end of the protein coding region of human importin α5 gene (GenBank: NM002264) was modified by adding a BamHI restriction site and a Kozak consensus sequence, ACC, prior to the translation start site (GGA TCC ACC ATG). To the C-terminal end of the gene a second BamHI site was added for further subcloning to a prokaryotic expression vector pGEX-2T(+) (GE Healthcare; GenBank: U13850.1) as described previously (25). Influenza A (A/PR8/34 H1N1) virus nucleoprotein (NP) gene (GenBank: NC002019.1) was subcloned to a baculoviral expression plasmid GST-pAcYM1 as described previously (26).

### Immunofluorescence and Antibodies

For visualization of intracellular location of filovirus VP24s, Huh7 cells were plated on coverslips and grown for 20h before transfection with expression plasmids for N-terminally HA-tagged VP24 gene. Twenty-four hours post transfection the cells were fixed with 4% paraformaldehyde at room temperature (RT) for 15 min, permeabilized with 0.1% Triton-X 100 in PBS for 5min and incubated for 1h at RT with primary mouse antibodies (anti-HA, Cell Signaling Technology) diluted in 3% BSA/PBS. After washing with 0.5% BSA/PBS the samples were labeled for 1h at RT with fluorescent Alexa Fluor 488 secondary anti-mouse antibodies (Thermo Fisher Scientific) diluted in 3% BSA/PBS. The coverslips were washed with 0.5% BSA/PBS and mounted with Moviol® 4-88ProLong Gold Antifade Mountant with DAPI (Thermo Fisher Scientific) in PBS on objective slides. Slides were imaged using Leica DFC7000 T fluorescent microscope with a 63x objective. Mitotracker staining of TAFV VP24 gene transfected cells was performed according to manufacturer’s instructions (MitoTracker® Red CMXRos, Cell Signaling Technology) followed by detection of VP24 protein as described above.

For the analysis of nuclear import of IRF3 Huh7 cells attached on coverslips in 12-well plates were transfected with EBOV wt and NLS-mutated VP24 expression constructs for 24 h. Cells were left unstimulated or stimulated by transfection of 10 μg of polyI:C per well with Lipofectamine 2000 according to manufacturer’s instructions. After 18h stimulation cells were fixed with 4% formaline in PBS for 15 min, permeabilized with 0.1% triton X-100 in PBS for 5 min and stained with mouse anti-HA and rabbit anti-IRF3 ^20^ antibodies followed by staining with Alexa-anti-mouse-568 and Alexa-anti-rabbit-488 and mounting (see above). The coverslips were visualized with Zeiss Axioimager microscope with 63x oil objective. Images were manually analyzed with Image J software and statistical differences between the groups were analyzed using Chi-square test.

### Reporter Gene Assays

HEK293 cells were grown on 96-well plates and transfected at 80-90% confluency with promoter-luciferase constructs (20ng/well), with wtRIG-I, ΔRIG-I or MDA5 expression plasmids (30ng/well) and filovirus VP24 or hepatitis C virus nonstructural protein (HCV NS3/4A) expression plasmids (3-30 ng/well). RSV-Renilla (50 ng/well) was included as an internal transfection efficacy control. Cells were harvested at indicated time points for Twinlite Dual Luciferase Reporter Gene Assay System (Perkin Elmer) according to the manufacturer’s instructions. Firefly luciferase results were normalized with Renilla luciferase values.

### Immunoblotting

For immunoblotting HEK293 cells were grown on 12-well plates. Cells were transfected with 500 ng of wtRIG-I, ΔRIG-I or MDA5 expression plasmids and with 250 ng of IRF3 expression plasmid. Filovirus VP24 expression plasmids were transfected in different amounts (200-2000 ng/well). After overnight incubation, cells were lysed on ice with Passive lysis buffer provided in Dual Luciferase Assay Kit (Promega), supplemented with Complete Protease Inhibitor Cocktail (Roche) and PhosStop Phosphatase Inhibitor Cocktail (Roche). Proteins were separated on in house 10% or 4–12% or Any kD SDS-PAGEs and transferred onto an Amersham Protran 0.2 μm nitrocellulose (GE Healthcare) or PVDF blotting membranes (Millipore). Immunoblotting was done using commercially available or in-house produced mouse anti-HA1.1 epitope tag (1:1000 dilution, BioLegend), mouse anti-GAPDH (1:700, 6C5, Santa Cruz Biotechnology), rabbit anti-IRF3 ^20^ (1:200), anti-P-IRF3 (4947, Cell Signaling Technology), rabbit anti-MDA5 (1:200) and rabbit anti-RIG-I (27) (1:200) antibodies. Anti-MDA5 antibodies were produced by immunizing rabbits 4 times with 50μg of E. coli produced MDA5 CARD domain (provided by Dr. J. Hiscott). Secondary antibodies were IRDye 800CW goat anti-rabbit IgG and IRDye 680RD goat anti-mouse IgG (LI-COR Biosciences). Membranes were scanned and analyzed with Odyssey Fc Imaging System (LI-COR Biosciences).

### Quantitative RT-PCR Analysis

Immortalized RIG-I^+/+^/MDA5^+/+^, RIG-I^-/-^/MDA5^+/+^, RIG-I^+/+^/MDA5^-/-^ and RIG-I^-/-^/MDA5^-/-^ MEF cells were harvested in 6-well plates after overnight stimulation either with synthetic EBOV ssRNA mimic or polyI:C. The cellular RNA was extracted using Trizol/RNeasy hybrid RNA extraction protocol (28). The relative expression levels of endogenous mouse Ifn-β mRNA was analyzed with quantitative RT-PCR and normalized to endogenous GAPDH mRNA levels with the ΔΔCt-method.

### Phylogenetical Analysis

**Figure 1:**
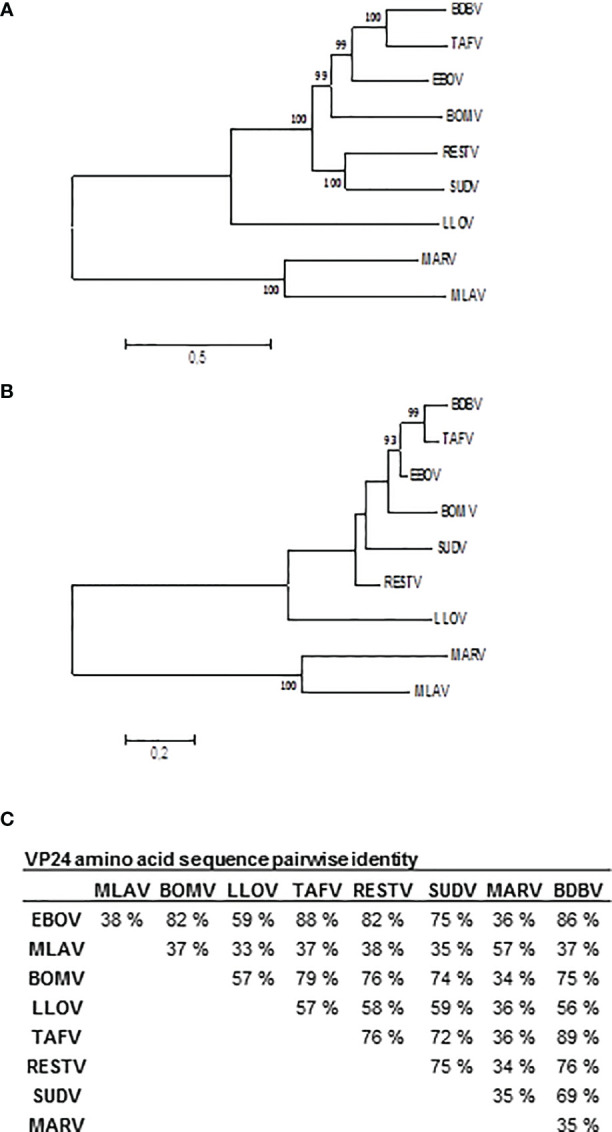
Phylogenetic analysis of complete genome **(A)** and VP24 amino acid sequences **(B)** of nine filoviruses. Sequences were obtained from Genbank: Bombali (BOMV; Accession number MF319185), Bundibugyo (BDBV; KC545394), Ebola (EBOV; KM233113), Reston (RESTV; KY798006), Sudan (SUDV; KC545389), Taï Forest (TAFV; KU182910), Lloviu (LLOV; NC016144.1), Marburg (MARV; NC001608.3), and Mengla virus (MLAV; KX371887) sequences were aligned with MUSCLE. Table **(C)** presents the amino acid sequence identities between filovirus VP24s. Phylogenetic analysis was carried out using maximum likelihood method based on the general time reversible (GTR) model for nucleotide sequences and general reverse transcriptase model for amino acid sequences with correction for gamma distribution to reconstruct the phylogenetic trees in MEGA 7 software platform. Evolutionary invariable sites were allowed in the GTR-model. The substitution models were chosen based on the model test in MEGA 7. One thousand bootstrap pseudoreplicates were used to estimate the reliability of the analysis.

To analyze the filovirus sequences, complete genomes or VP24 sequences of the viruses listed in **Figure 1** were aligned using Multiple Sequence Comparison by Log Expectation (MUSCLE). Best model for the description of the phylogenetic relationships was estimated with Molecular Evolutionary Genetics Analysis Computing Platform 7 (MEGA 7) and the phylogenetic trees were constructed in the same platform by maximum likelihood approach using General Time Reversible model with gamma distribution and possibility of evolutionary invariability for some sites.

### Importing Binding Assays

Human importin α5 was expressed in *E. coli* as a GST fusion protein in BL21 cells under isopropyl-1-thio-D-galactopyranoside induction (26). Bacteria were lysed in 50 mm Tris-HCl buffer, pH 7.4, 150 mm NaCl, 5 mm EDTA, 1% Triton X-100 (IP buffer) with 5 mg/ml lysozyme (Sigma-Aldrich Co.) for 30 min at room temperature, briefly sonicated, and clarified by Eppendorf centrifugation (13,000 rpm, 5 min). Bacterial cell extract, containing GST-importin α5, was allowed to bind to Glutathione Sepharose 4B beads (GE Healthcare) at +4 °C in IP buffer for 60 min followed by washing for two times. The purity and quantity of each fraction were verified with Coomassie Blue staining on 12% SDS-PAGE. To produce GST influenza A virus NP fusion protein, *Sf9* cells were infected for 72 h with GST-NP gene expressing baculoviruses as described elsewhere (26). The cells were collected, whole cell extracts were prepared, and GST-NP fusion protein was bound to Glutathione Sepharose 4B beads as described above. To produce VP24 proteins for importin binding assays, HEK293 cells on 6-well plates, were transiently transfected with pEBB-HA-BOMV VP24, pEBB-HA-LLOV VP24, pEBB-HA-RESTV VP24, pEBB-HA-SUDV VP24, pEBB-HA-TAFV VP24, or pcDNA3.1(+)/myc-His-EBOV VP24 expressing gene constructs using TransIT-LT1 transfection reagent (Mirus Bio LCC) according to the manufacturer’s instructions. After 24 h, the cells were collected, lysed in IP buffer by suctioning the samples through a 25 G needle, followed by centrifugation (10,000 x g, +4 ^0^C, 5 min). For pull-down experiments, soluble cellular protein samples were bound to 25 μL of Glutathione Sepharose-immobilized GST and GST-fusion proteins at +4°C for 1 h and washed three times with IP buffer. Those filovirus VP24 proteins that could not be efficiently produced or extracted from transfected HEK293 cells were *in vitro*-translated as radioactively labeled proteins (TnT® Quick Coupled Transcription/Translation Systems; Promega, Madison) BDBV and MLAV VP24 cDNAs in pcDNA3.1+ plasmid (Thermo Fisher Scientific) and MARV VP24 cDNA in pcDNA3.1+/myc-His B plasmid were [^35^S]-Met/Cys-labeled (Easy Tag^TM^ Express Protein Labeling Mix, PerkinElmer) and allowed to bind to Sepharose-immobilized GST and GST-fusion proteins as described above. For analysis of GST or GST-fusion protein-bound proteins, Sepharose beads were dissolved in Laemmli sample buffer, and the proteins were separated on 12% SDS-PAGE. Samples from transiently transfected HEK293 cells were transferred onto PVDF membranes (Millipore,) followed by staining with primary anti-HA1.1 and secondary anti-mouse HRP antibodies (Dako). Gels with [^35^S]-labeled proteins were fixed and treated with Amplify reagent (Amersham Biosciences). Autoradiography was performed using HyperMax films (Amersham Biosciences).

## Results

### Phylogenetic Analysis of Filovirus VP24s

Analysis of the filovirus complete genome and VP24 gene sequences indicate that the species arrange phylogenetically similarly when complete genomes (**Figure 1A**) and the VP24 genes (**Figure 1B**) are compared. The species belonging to the genus *Ebolavirus* were 74-90% identical in their VP24 amino acid sequences while the identity with LLOV was 56-59%, with MARV 34-36% and with MLAV 37-57% (**Figure 1C**).

### Cloned Filovirus VP24 Genes Are Efficiently Expressed in Huh7 Cells

**Figure 2:**
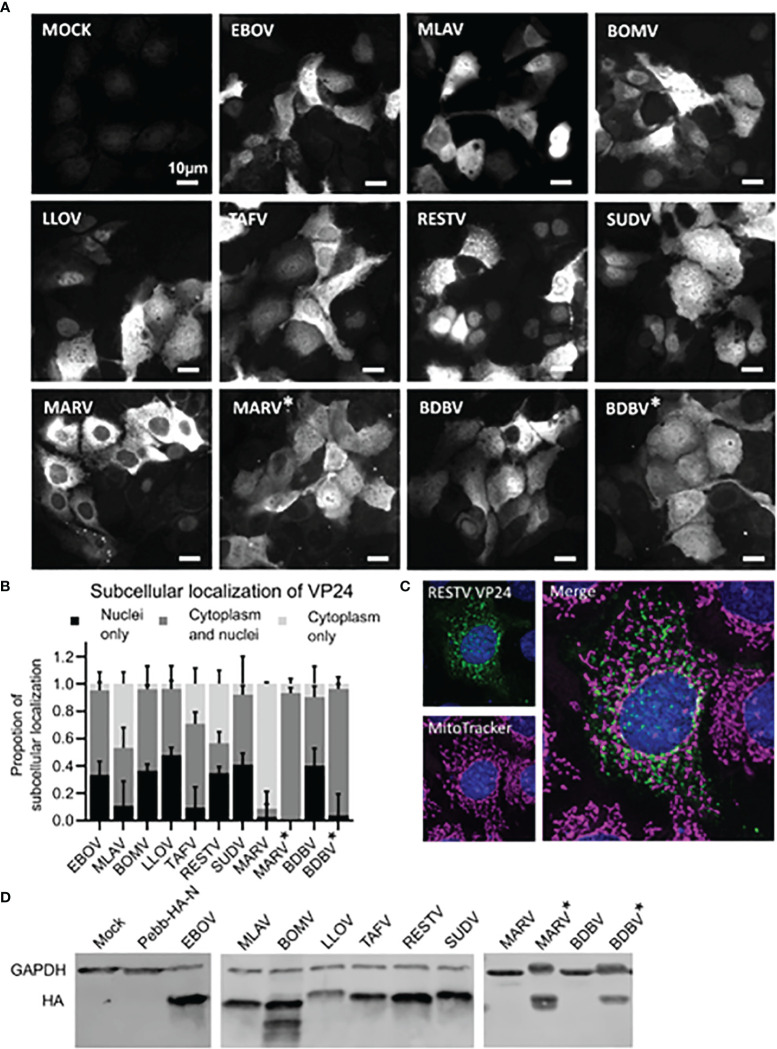
Subcellular location of nine filovirus VP24 proteins. Huh7 cells were transfected for 24 hours with different filovirus VP24 expression plasmids (2.5µg/well in 6-well plates). For imaging purposes cells were fixed and permeabilized followed by labeling with anti-HA antibody and fluorescent secondary antibodies. **(A)** Representative images of filovirus VP24 expressing Huh7 cells. BDBV* and MARV* refer to codon optimized expression constructs of the respective genes due to the low or undetectable expression of the original gene sequences in Western blotting. **(B)** Quantification of subcellular localizations of different filovirus VP24s. The quantitation was based on 3 experimental repeats in each of which 100-200 cells per VP24 were analyzed, scale bar 10µm, error bars are standard error of means. **(C)** Representative image of RESTV VP24 expressing Huh7 cell stained with anti-HA antibody (for VP24) and MitoTracker® stain is shown. **(D)** Western blot analysis of indicated VP24 proteins and controls in HEK293 cells. The cells were transfected with 1200 ng in 12-well plate for 24 h, protein samples were collected and 20% of total cellular proteins were separated on 10% SDS-PAGE, transferred onto PVDF membranes and detected with anti-HA and anti-GAPDH antibodies followed by Alexa-anti-mouse-568.

The expression of VP24 proteins from the VP24-pEBB-HA-N plasmids was confirmed by immunofluorescence. Codon optimized constructs for BDBV and MARV VP24 were also included. Immunofluorescence analysis showed that the expression of VP24 proteins with N-terminal HA-tag is efficient in transfected Huh7 cells (**Figure 2A**). Based on the immunofluorescence imaging the subcellular localization of different VP24s were quantified in 3 individual experiment in which 100-200 cells were counted for each VP24 protein expressing cells (**Figure 2B**). Interestingly, the majority of VP24s studied were predominantly expressed both in the cytoplasm and nucleus (EBOV 95%, MLAV 53%, BOMV 96%, LLOV 96%, TAFV 71%, RESTV 56%, SUDV 92%, BDBV 50% and BDBV* 92%; * refers to codon optimized protein) with the remaining VP24 positive cells being either solely in the nucleus or in the cytoplasm (**Figure 2B**). The authentic MARV VP24 gene encoded protein was predominantly expressed in the cytoplasm (91%), but the codon optimized version of MARV* VP24 was predominantly both in the nucleus and cytoplasm (93%; **Figure 2B**).

Interestingly, VP24 of RESTV formed granule-like structures in the cell cytoplasm resembling mitochondrial structures. This expression pattern was clearly different from the other VP24 proteins. Since RIG-I and MDA5 signaling takes place on mitochondrial membranes, we performed a colocalization analysis of RESTV VP24 and mitochondria by staining the cells for RESTV VP24 and mitochondria. RESTV VP24 formed distinct granular structures that were clearly separate from mitochondria (**Figure 2C**).

To ensure that the expression constructs are encoding correct size proteins HEK293 cells were transfected for 24 h with different expression plasmids followed by analysis of VP24 protein expression by immunoblotting. All authentic VP24 gene encoded proteins, except those for MARV and BDBV VP24 proteins, were clearly visible. The codon optimized constructs of MARV and BDBV VP24 were expressed in similar quantities as other VP24s (**Figure 2D**).

### *Ebolavirus* Genus and Lloviu VP24 Proteins Directly Interact With Human Importin α5

**Figure 3:**
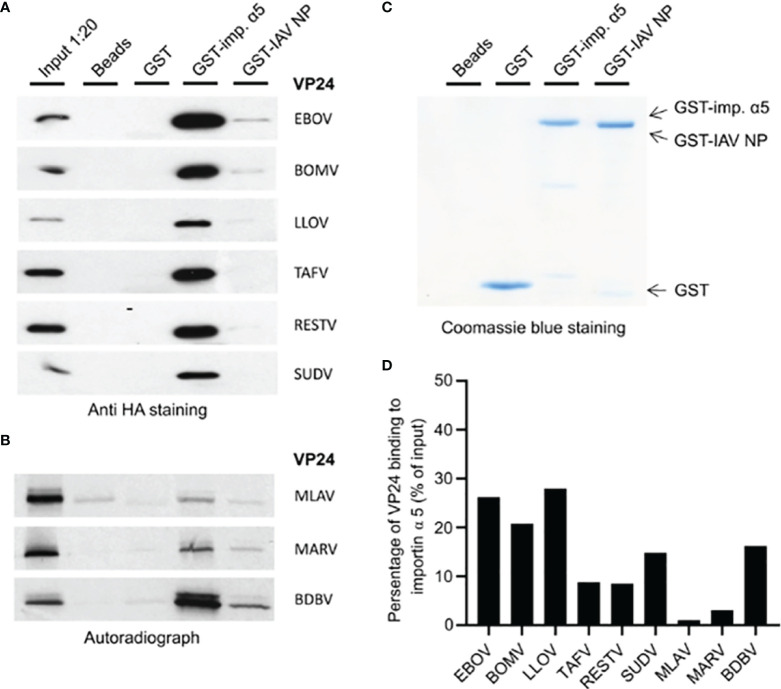
Binding of filovirus VP24 proteins to human importin α5. **(A)** HEK293 cells were transiently transfected in a 6-well format with BOMV, RESTV, SUDV, TAFV, EBOV or LLOV VP24 expression plasmids (4 μg/well). At 24 h post-transfection, soluble cell extracts were prepared, and proteins in cell extracts were allowed to bind to empty beads or bead-immobilized GST, GST-importin α5, and influenza A virus GST-nucleoprotein (GST-IAV-NP) as another nonspecific binding control. Bound proteins were separated on 12% SDS-PAGE, and the presence of VP24 proteins were analyzed by immunoblotting with anti-HA antibodies. Control lanes include ca. 5% of the amount of input cell extracts for different VP24 proteins (input 1:20). **(B)** [35S]Met/Cys-labeled and *in vitro*-translated BDBV, MARV and MLAV VP24 proteins were allowed to bind to beads alone and immobilized GST, GST-importin α5 and GST-IAV-NP. Bound proteins were separated on 12% SDS-PAGE, and the presence of VP24 proteins were analyzed by autoradiography. Control lanes show *in vitro*-translated BDBV, MARV and MLAV VP24 proteins, and each lane represents 1:20 of the amount of reticulate lysate that was used in each binding experiment. **(C)** Coomassie Blue-stained gel is shown to visualize the amount of Glutathione Sepharose-bound GST, GST-Importin α5, and GST-IAV-NP. **(D)** Quantitation of relative VP24 binding efficacy to GST-importin α5. VP24 band intensities in immunoblots were scanned and the percentage of GST-importin α5 bound VP24 of the input is shown. The percentage was obtained by dividing the intensity of bound VP24 signal with the input signal multiplied by 20 (loading included 1:20 of the amount of VP24 in binding experiments).

In our previous study, we suggested that binding of VP24 to importin α molecules is required for VP24-dependent inhibition of IFN-λ1 gene expression ^10^. Also, to inhibit IFN signaling EBOV VP24 is known to require an NLS/importin α binding site. A previous study has shown that the sequence differences in the main three clusters (clusters 1-3; **Supplementary Figure 2**) of this binding site dictate the differences in VP24 binding to karyopherin alpha (KPNA) (also called importin α5) (29). We analyzed the amino acid sequences of VP24s of these three clusters in different filovirus VP24s and observed that the whole *Ebolavirus* genus (6 viruses) and LLOV VP24 show high sequence identity in protein clusters 1 and 3 while MARV and MLAV VP24s differ from other filovirus VP24s in all 3 clusters (**Supplementary Figure 2**). Since filovirus VP24s were also located in the cell nucleus, we analyzed whether they were binding to importin α5. GST pull-down experiments with VP24 expression plasmid transfected cell extracts or for low-expressing/aggregating *in vitro* translated VP24 species indicated that all VP24 proteins bound to importin α5 with MARV and MLAV VP24 showing very weak binding (**Figure 3**). The data suggests that importin α/β pathway is likely regulating the nuclear import of all filovirus VP24 proteins.

### EBOV Genomic RNA Mimic Activates Both RIG-I and MDA5 Pathways in Mouse Embryonal Fibroblast Knock-Out Cell Lines

**Figure 4:**
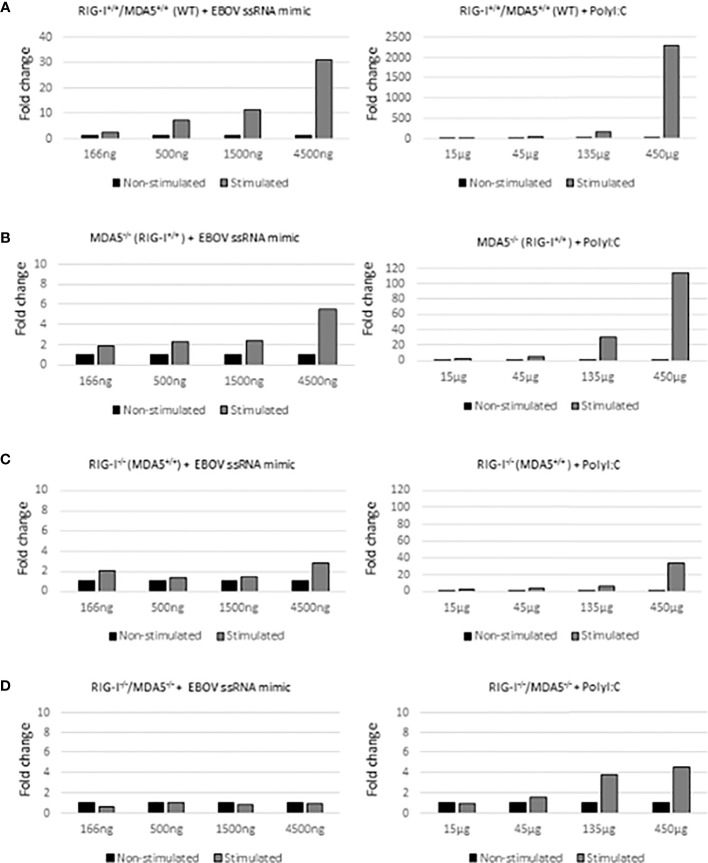
Regulation of Ifn-β gene expression in RIG-I and MDA5 defective mouse cell lines by EBOV ssRNA mimic. Immortalized mouse embryonic fibroblast (MEF) cells were stimulated with EBOV ssRNA mimic construct or polyI:C and after overnight incubation the cells were harvested for RT-PCR. The expression levels of endogenous Ifn-β mRNA were analyzed and normalized with Gapdh levels. In the X-axis there is an increasing amount of either EBOV ssRNA mimic (166 to 4500 ng/well) or polyI:C (15 to 450 μg/well) and Y-axis indicates the fold change in Ifn-β mRNA expression. Note the differences in ssRNA and polyI:C amounts. Relative expression of Ifn-β mRNA is shown in wild type MEFs [panel **(A)** RIG-I^+/+^/MDA5^+/+^], MDA5 defective [**(B)** RIG-I^+/+^/MDA5^-/-^], RIG-I defective [**(C)** RIG-I^-/-^/MDA5^+/+^], and RIG-I/MDA5 double knockout cells [**(D)** RIG-I^-/-^/MDA5^-/-^]. A representative experiment out of 3 is shown.

RIG-I and MDA5 act as the main PRRs in early innate immune response (30). To investigate their differential role in regulating type I interferon genes (Ifn-λ1 gene is defective in mice) in response to stimulation with genomic EBOV ssRNA mimic (described in **Supplementary Figure 1**), RIG-I^+/+^/MDA5^+/+^, RIG-I^-/-^/MDA5^+/+^, RIG-I^+/+^/MDA5^-/-^ and RIG-I^-/-^/MDA5^-/-^ MEF cells were stimulated with increasing amounts of EBOV ssRNA mimic (**Figures 4A–D**, left column). As a positive control, polyI:C was used (**Figures 4A–D**, right column). The expression of endogenous Ifn-β mRNA was measured with qRT-qPCR and the signal was normalized using an endogenous control, Gapdh mRNA. As expected, polyI:C stimulated RIG-I^+/+^/MDA5^+/+^ MEF cells showed a dose-dependent expression of Ifn-β mRNA (**Figure 4A**, right column). Also, EBOV ssRNA mimic induced Ifn-β mRNA expression in RIG-I^+/+^/MDA5^+/+^ cells in a dose-dependent fashion (**Figure 4A**, left column). Interestingly, a clear difference in Ifn-β mRNA expression was detected in RIG-I^-/-^/MDA5^+/+^ and RIG-I^+/+^/MDA5^-/-^ MEF cells with a functional RIG-I molecule showed a better response to EBOV ssRNA mimic stimulation as compared to RIG-I defective, MDA5-positive cells (**Figures 4B, C**, left column). Although both RIG-I and MDA5 pathways appeared to be activated by EBOV RNA, Ifn-β mRNA expression was more strongly dependent on the RIG-I pathway (**Figures 4B, C**, right column). As expected, the MEF cell line lacking both RIG-I and MDA5 showed no increased Ifn-β gene expression (**Figure 4D**).

### Type I and III Interferon Promoters Are More Strongly Activated by the RIG-I Pathway Than the MDA5 Pathway

**Figure 5:**
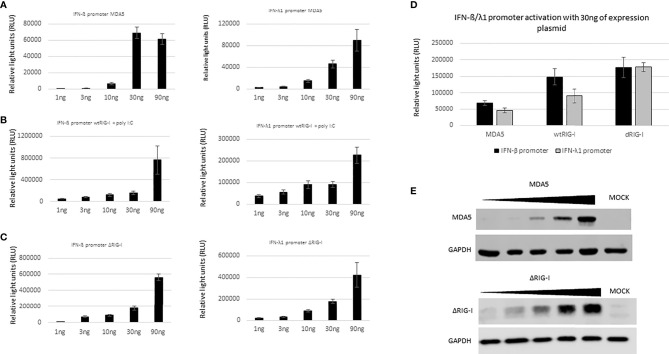
Comparative ability of MDA5 and RIG-I to stimulate IFN-β and IFN-λ1 promoter activation. **(A)**. HEK293 cells were transfected in a 96-well format with expression plasmids for MDA5, wtRIG-I and ΔRIG-I (from left to right 1, 3, 10, 30, 100 ng/well) and reporter plasmids IFN-β or IFN-λ1-promoter-luciferase (30 ng/well) and RSV-Renilla (50 ng/well). wtRIG-I expression plasmid transfected cells were stimulated with polyI:C (10 μg/ml) at 4 h post-transfection. After 24 h incubation luciferase activities were measured. **(B)** Comparative presentation of luciferase activity obtained with 30ng/well of expression plasmids. Data obtained from panel **(A)** A representative experiment of 3 is shown. **(C)** HEK293 cells were transfected with increasing amounts of MDA5 and ΔRIG-I expression plasmids in a 12-well format (10, 30, 100, 300, 900 ng/well) for 24 h. Cells were collected and 20% of cell extracts were separated on 10% SDS-PAGE, transferred to PVDF membranes and stained with rabbit anti-MDA5 and anti-RIG-I antibodies followed by goat anti-rabbit IRDye 800CW.

It is known that RIG-I and MDA5 have common features but they also differ from each other (31). RIG-I preferentially recognizes small 5’-phosphorylated ss/dsRNA molecules while MDA5 is stimulated by longer non-5’-phosphorylated RNA molecules. To compare the relative stimulatory activity of RIG-I and MDA5 as activators of type I and III interferon promoters, HEK293 cells were transfected with increasing amounts of wtRIG-I, ΔRIG-I, (constitutively active form of RIG-I) and MDA5 expression plasmids together with IFN-β or IFN-λ1-promoter-luciferase reporter plasmids. Since wtRIG-I is not constitutively active, the cells needed to be additionally stimulated with polyI:C. As shown in **Figure 5**, an increase in IFN-β and IFN-λ1 promoter activation is dose-dependently following the increasing amounts of wtRIG-I+polyI:C, MDA5 and ΔRIG-I (**Figure 5A**), the increasing expression of the latter two molecules confirmed by immunoblotting (**Figures 5A–C**). Of note, in high plasmid amounts wtRIG-I or ΔRIG-I expressing cells showed up to 10-fold higher IFN-β-Luc and 2 to 4-fold higher IFN-λ1-Luc promoter activities as compared to those with MDA5 stimulation. Based on these results, we chose the amount of 30ng of expression plasmids (**Figures 5B, D**) for further studies on the potential inhibitory effects of different filovirus VP24s on RIG-I and MDA5 signaling pathways.

### Filovirus VP24 Protein Differentially Inhibits IFN-λ1 and IFN-β Promoter Activation

**Figure 6:**
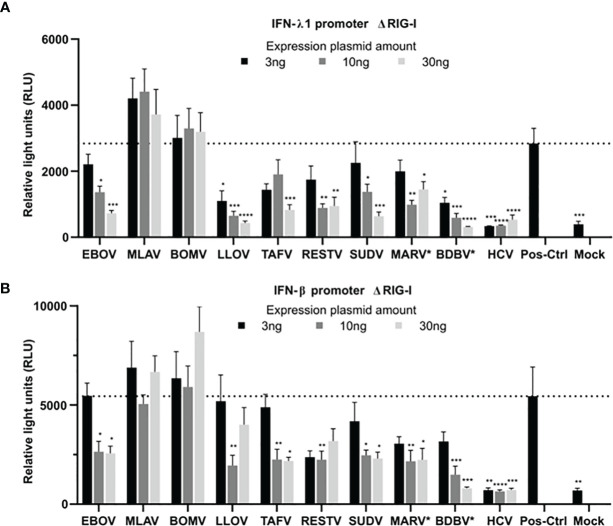
Inhibitory effects of filovirus VP24 proteins on RIG-I induced IFN-λ1 and IFN-β gene expression. HEK293 cells were transfected with increasing amounts (3, 10 or 30 ng/well) of filovirus VP24 expression plasmids (*codon optimized) together with expression plasmid ΔRIG-I and reporter plasmids IFN-λ1 **(A)** or IFN-β **(B)** promoter-luciferase (30 ng/well) and RSV-Renilla (50 ng/well). Cells were harvested after overnight incubation and measured for the luciferase activity. Controls include ΔRIG-I, promoter-reporter plasmids and HCV NS3/4 plasmids (3, 10 or 30 ng/well). Luciferase values were normalized with RSV-Renilla expression control. The bars represent the mean values of three independent experiments with three replicates (n=9). Error bars indicate standard errors. Statistical differences were calculated by ordinary one-way ANOVA Dunnett’s multiple comparisons test with a single pooled variance. Each column value was compared to the positive control. P values, *p = < 0.05, **p = < 0.005, ***p = < 0.0005, ****p = < 0.0001.

Next, we systematically analyzed the potential inhibitory effects of filovirus VP24 proteins on RLR stimulated IFN-λ1 and IFN-β promoter activation. HEK293 cells were transfected with the expression plasmids for ΔRIG-I, IFN-λ1 or IFN-β-promoter-luciferase reporter, RSV-Renilla and increasing amounts of different filovirus VP24 expression constructs (only codon optimized versions of MARV and BDBV were used). Overexpression of ΔRIG-I was included as a positive control and HCV NS4/3A as a negative control since it is known to cleave the MAVS protein and release it from the mitochondrial membrane efficiently abolishing RIG-I signaling (24). IFN-λ1 and IFN-β promoter activity was measured after overnight incubation and luciferase values were normalized with Renilla luciferase values. As expected, HCV NS3/4A strongly inhibited IFN-λ1 and IFN-β promoter activation (**Figures 6A, B**). VP24 proteins of EBOV, LLOV, TAFV, RESTV, SUDV, MARV and BDBV dose-dependently inhibited the activation of the RIG-I pathway. The level of inhibition appeared to be more pronounced for IFN-λ1 promoter. In contrast, MLAV and BOMV VP24 proteins appeared to show no inhibition of ΔRIG-I induced IFN-λ1 or IFN-β promoter activation, not even with the highest amounts of VP24 expression plasmids.

### EBOV VP24 Efficiently Inhibits Both the RIG-I and MDA5 Pathways

**Figure 7:**
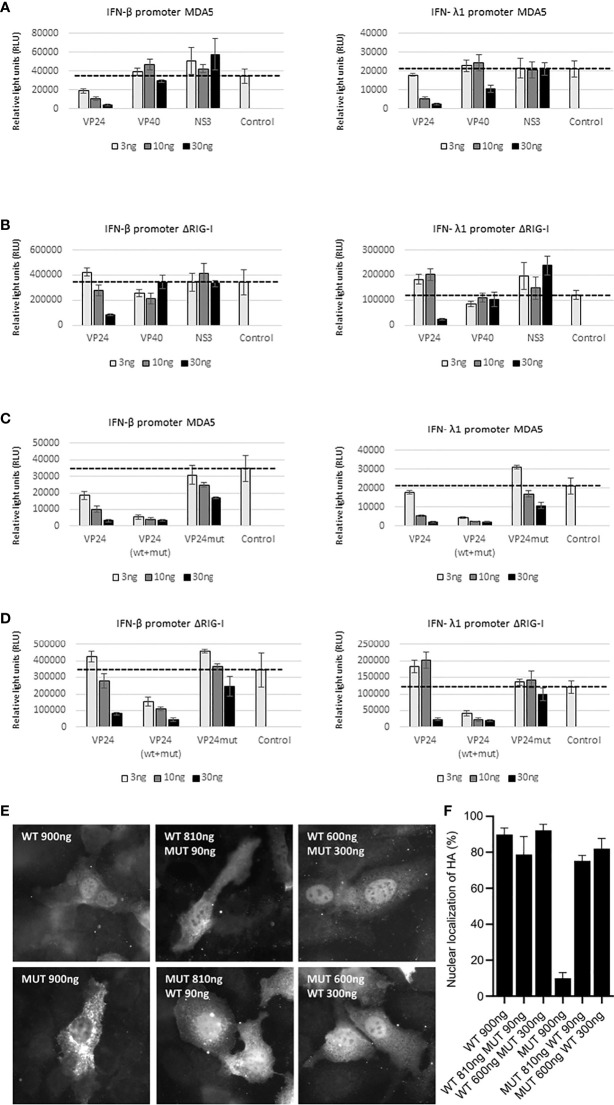
Inhibition of MDA5 and RIG-I induced IFN-β and IFN-λ1 gene expression by wild type and NLS-mutated EBOV VP24. **(A)** HEK293 cells were transfected with increasing amounts (3, 10, 30 ng/well in 6-well plates) of EBOV VP24 and VP40 and Zika virus NS3 expression constructs. Positive control refers to MDA5 induced promoter activity in the absence of viral proteins. **(B)** Experimental setting as in panel **(A)**, but promoter activation is induced by ΔRIG-I. **(C, D)** HEK293 cells were transfected with increasing amounts of EBOV VP24 or NLS-mutated VP24 (VP24mut) expression plasmids (3, 10 or 30ng/well) together with an expression plasmid ΔRIG-I and reporter plasmids IFN-β/IFN-λ1 -promoter-luciferase (30 ng/well) and RSV-Renilla. VP24 (wt+mut) refers to combinations of wt and NLS-mutated VP24 expression plasmids (3+27 ng, 10+20 ng and 30+0 wt-NLS mutant plasmid ratios). Cells were harvested at 24 h after incubation and luciferase values were normalized with RSV-Renilla control. The experiment was repeated three times and values represent the mean values. Error bars indicate standard errors of the mean. Positive controls refer to MDA5 or ΔRIG-I induced promoter activity without VP24 expression plasmids.

RIG-I and MDA5 are essential molecules in regulating the production of type I and III interferons (31). Previously we have shown that EBOV VP24 efficiently inhibits RIG-I dependent IFN-λ1 promoter activation and this activation requires an intact NLS/importin α interaction site of VP24 (10). Since both RIG-I and MDA5 play critical roles in host innate immune responses, we analyzed the effect of EBOV VP24 protein separately on RIG-I and MDA5 dependent IFN gene promoter activation. HEK293 cells were transfected with ΔRIG-I or MDA5 expression constructs together with IFN-β or IFN-λ1-promoter-luciferase reporter constructs. As negative transfection controls, EBOV VP40 and Zika virus (ZIKV) NS3 expression constructs with no inhibitory effect on the RIG-I pathway were used (32). As shown in **Figures 7A, B**, EBOV VP24 strongly and dose-dependently inhibited IFN-β and IFN-λ1 promoter activation downstream of RIG-I and MDA5 pathways while no inhibition was seen by EBOV VP40 and ZIKV NS3 proteins (**Figures 7A, B**). It was of interest that EBOV VP24 almost completely inhibited MDA5 induced IFN promoter activity (**Figure 7A**), while the activity induced by RIG-I was inhibited to a lesser extent. This may be explained by the higher stimulatory activity of RIG-I compared to that of MDA5 and thus higher levels of VP24 would be needed to fully inhibit the RIG-I pathway.

Previously, we demonstrated that EBOV VP24 that has its NLS/importin α interaction site mutated, rendering the protein preferentially cytoplasmic, shows strongly decreased inhibition of ΔRIG-I induced IFN-λ1 promoter activation (10). Here we transfected HEK293 cells with MDA5 (**Figure 7C**) or ΔRIG-I (**Figure 7D**) expression plasmids together with IFN-β or IFN-λ1-promoter-luciferase reporter and with EBOV wtVP24 or NLS-mutated VP24. Increasing amounts of wtVP24 efficiently inhibited RIG-I and MDA5 induced IFN promoter activation, while the NLS-mutated VP24 was almost devoid of this inhibitory activity (**Figures 7C, D**). Interestingly, mixing wt and NLS-mutated VP24 expression plasmids (total amount of DNA 30 ng) efficiently inhibited RIG-I and MDA5 induced IFN promoter activation suggesting that wtVP24 - NLS-mutated VP24 dimers likely enter the nucleus in sufficient amounts to inhibit the activation of IFN promoters. Even small amounts of wtVP24 (10%) mixed with NLS-mutated VP24 expression plasmid (90%) enabled VP24 to be efficiently targeted into the nucleus (**Figures 7E, F**). Our data shows that EBOV VP24 has to be located in the nucleus to efficiently inhibit RIG-I and MDA5 regulated IFN gene expression.

### Filovirus VP24s Do Not Inhibit the Phosphorylation of IRF3 and EBOV VP24 Does Not Inhibit the Nuclear Import of Activated IRF3

**Figure 8:**
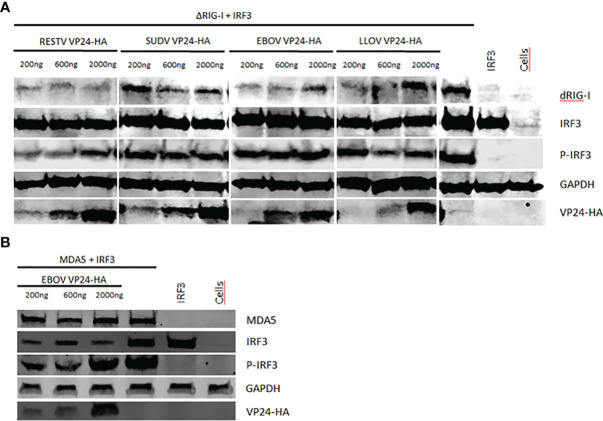
The effect of RESTV, SUDV, EBOV and LLOV VP24 on RIG-I and MDA5 activated IRF3 phosphorylation. HEK293 cells were transfected with increasing amounts of expression plasmids for VP24s (200–2000 ng/well in 6-well plates) together with **(A)** ΔRIG-I or **(B)** MDA5 and IRF3 expression constructs (30 ng/well). After overnight incubation cells were harvested, separated on SDS-PAGE, transferred to nitrocellulose membranes and stained with specific antibodies against different proteins as indicated in the figure. GAPDH detection with anti-GAPDH antibody was used as a loading control. P-IRF3 refers to activated, phosphorylated form of IRF3. The experiment was repeated twice with similar results. A representative experiment is shown.

In our previous study we showed that EBOV VP24 does not inhibit the phosphorylation of IRF3 (10). Since there was a clear dose-dependent inhibition of activation of the RIG-I pathway in most filovirus VP24 expressing cells, we analyzed whether some of these proteins had an ability to inhibit RIG-I or MDA5 regulated IRF3 phosphorylation (**Figure 8A**). To study this, HEK293 cells were transfected with ΔRIG-I (**Figure 8A**) or MDA5 (**Figure 8B**) expression plasmids together with IRF3 and increasing amounts of RESTV, SUDV, EBOV and LLOV VP24 expression plasmids. As shown in **Figure 8A**, in the presence of filovirus VP24s, IRF3 is efficiently phosphorylated and none of the four VP24 proteins exhibited a detectable inhibition of IRF3 phosphorylation. We also analyzed the phosphorylation of IRF3 downstream of MDA5, since the EBOV RNA mimic was found to activate both pathways. Like in ΔRIG-I stimulation experiments, EBOV VP24 was not able to inhibit MDA5 stimulated IRF3 phosphorylation (**Figure 7B**).

**Figure 9:**
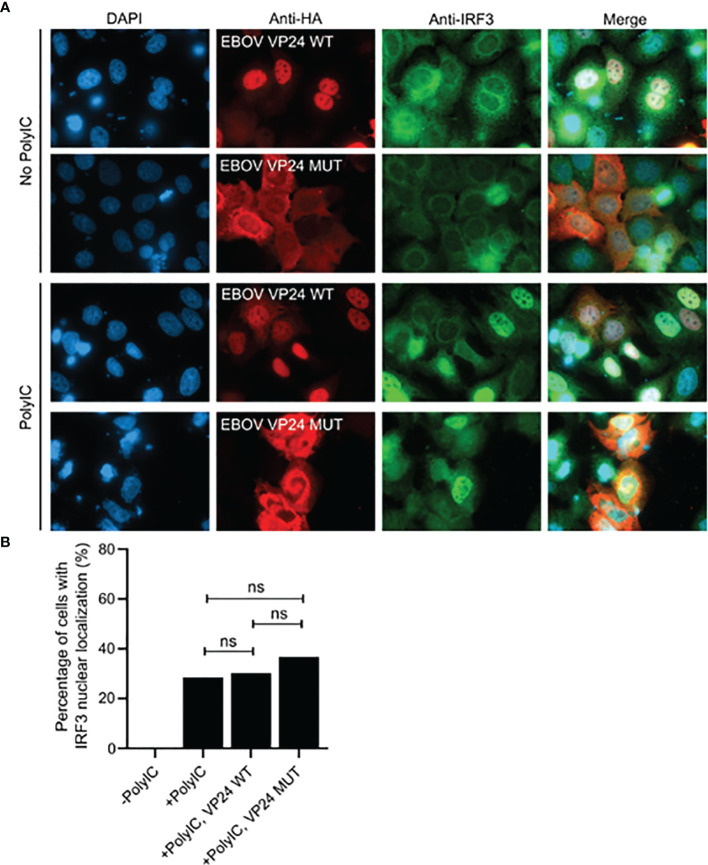
The effect of EBOV VP24 on nuclear import of IRF3. Huh7 cells we transfected with EBOV VP24 expression plasmids for 24 h (1200ng of plasmid per 12-well), followed by stimulation of the cells with 10µg of polyI:C for 18 h. **(A)** Fixed and permeabilized cells were stained with anti-HA and anti-IRF3 antibodies and fluorescent secondary antibodies. **(B)** Nuclear localization of IRF3 was quantified. Bars represent the average of IRF3 nuclear localization in each sample. From each variable 100 to 110 cells were analyzed and statistical analysis was performed using Chi-square test. ns, non-significant.

In order to mechanistically reveal this observation further we analyzed whether EBOV wtVP24 or mutVP24 can interfere with the nuclear import of activated IRF3. Huh7 cells were selected for these analyses due to their good cellular morphology and distinct intracellular location of VP24s (**Figure 2**). EBOV VP24 expressing cells stimulated with polyI:C showed that IRF3 accumulation in the nucleus occurred equally efficiently in control cells and in wtVP24 and mutVP24 expressing cells (**Figure 9**). The data indicates that VP24 is not inhibiting the nuclear import of activated IRF3.

## Discussion

A vast majority of filovirus research has been focusing on EBOV due to its recent outbreak in West Africa which led to a very high number of deaths. A subsequent epidemic in the Democratic Republic of Congo further emphasized the urgent need for understanding the transmission and pathogenesis of Ebola and other filoviruses. EVD and Marburg virus disease (MVD) are characterized as an acute disease with rapid and uncontrolled viral replication and suppression of host antiviral responses, mainly downregulation of the production of type I and III interferons (33). Uncontrolled inflammatory responses in filovirus infection may also lead to excessive production of immunological mediators, better known as a cytokine storm, causing distinct symptoms of EVD and MVD (34). Currently there are six filoviruses that have been recorded as human pathogens (35). In this study, we systematically analyzed the effects of nine filovirus VP24 proteins on RIG-I and MDA5 stimulated type I and III interferon promoter activation. We also demonstrated that a synthetic EBOV ssRNA mimic stimulated both RIG-I and MDA5 pathways and both pathways were inhibited by most filovirus VP24s. The inhibition occurred downstream of IRF3 and all VP24s capable of inhibiting IFN promoter activation were located in the cell nucleus. Nuclei targeted EBOV VP24 was also inhibiting the nuclear accumulation of activated IRF3. However, nuclear localization as such was not sufficient for a VP24 to inhibit IFN gene expression (e.g. MLAV and BOMV).

Our current study was initialized by first investigating the possible differences in filovirus genomes covering four genera (*Ebolavirus, Marburgvirus, Cuevavirus* and *Dianlovirus*) and their VP24 proteins. Previously, it has been shown that EBOV VP24 protein can inhibit type I and III interferon promoter activation (10). The phylogenetic analysis between different filoviruses revealed quite large differences between the genomes. Also, the difference between the EBOV and MARV viruses was significant showing only as little as 34% of amino acid sequence identity. A recent study identified two new filovirus species in fish showing that filoviruses have relatives in aquatic vertebrates and suggests for a possibility that filoviruses have differentiated even further (36).

We analyzed the intracellular localization of filovirus VP24 proteins and interestingly found that VP24s localize into different cellular compartments with varying efficiency, however, all VP24s were found both in the cell nucleus and cytoplasm. MARV VP24 behaved somewhat differently, in low expression levels it was cytoplasmic, but high expression induced by codon optimized MARV VP24 expression construct rendered the protein nucleo-cytoplasmic. Accumulation of different filovirus VP24s in the nucleus correlated very well with their interaction with importin α5; all six *Ebolavirus* VP24s and the Lloviu VP24 bound importin α5 and they were also primarily found in the nucleus. It was of interest that MARV and MLAV VP24s that are genetically more distant from *Ebolavirus* VP24s bound importin α5 extremely weakly, however, when the expression level was high, these proteins were yet targeted into the nucleus. MARV VP24 was also able to inhibit IFN promoter activation.

Due to the dominant role of RLRs in filovirus infections we produced synthetic genomic EBOV RNA mimic to activate MEF cells that were either RIG-I or MDA5 defective and compared them to the wild type cells and RIG-I/MDA5 double knock-out cells. Stimulation of cells with EBOV ssRNA mimic showed that both RIG-I and MDA5 were involved in IFN gene expression, but the RIG-I pathway appeared to be the dominating one. The dominant role of RIG-I in EBOV ssRNA induced responses may be due to the overall higher signaling activity of RIG-I compared to that of MDA5. RIG-I and MDA5 detect somewhat different viral genome structures, but yet these PRRs may work synergistically to recognize different elements of the same viral genome (37).

Since the difference in VP24 is up to 26%, it is expected that there may be differences in their ability to mediate interferon antagonism and downregulate IFN stimulated genes. Typically EBOV VP24 has been shown to inhibit type I and III interferon gene expression and also the nuclear import of IFN-induced phosphorylated and dimerized STAT1 and STAT2 (10, 29). Also, EBOV VP24 has been shown to interact with several importin α subtypes, especially importins α5 and α6 ^9,10^. It is commonly known that host innate immune responses to filovirus infection is largely dependent on both type I and III IFNs (IFN-α/β/λ). Previously, it has been shown that EBOV VP24 strongly inhibits IFN gene expression (10, 12). In the present study we extended these observations to cover other *Filoviridae* family members and all studied filovirus VP24 except those of MLAV and BOMV clearly inhibited IFN promoter activation. IRF3 phosphorylation and nuclear import of IRF3, in the case of EBOV VP24, was not affected by inhibitory VP24 proteins stimulated by ΔRIG-I and MDA5. Both pathways seemed to be specifically inhibited by those filovirus VP24 molecules that were able to downregulate RLR signaling.

In the case of EBOV VP24, the ability to bind to importin α is functionally important since the NLS mutant of EBOV VP24 did not efficiently inhibit RIG-I induced IFN gene expression (10). These observations were further extended here and we showed that both RIG-I and MDA5 pathways were inhibited by EBOV wtVP24 while the inhibitory activity of NLS mutated VP24 was very weak. However, if wt and NLS mutant VP24 plasmids were mixed even low amounts of wtVP24 was sufficient to trigger wt-mutVP24 heterodimer import into the nucleus and mediate inhibitory activity on IFN promoter activation. This suggests that nuclear localization of VP24 is essential for its inhibitory activity on RLR signaling.

In most part, our data on the inhibitory activity of filovirus VP24 of IFN gene expression is well in line with previous publications (34, 38). Previously, it has been shown that EBOV and RESTV inhibit type I interferon production (39). MLAV VP24 has been shown not to inhibit IFN-induced gene expression (38 )which is well in line with the results of the present study. Some differences, such as the ability of MARV VP24 to inhibit IFN promoter activation between our and published data can be due to differential experimental conditions and MARV VP24 protein expression level. Our study provides a systematic comparison of all available mammalian-infecting filovirus VP24 proteins on IFN-λ1 and IFN-β promoter activation and we show that seven out of nine filovirus VP24s are efficiently inhibiting IFN promoter activation. Among these filoviruses the only filovirus that has not so far been seen as a human pathogen is LLOV. Another interesting finding is the role of RIG-I and MDA5 pathways in filovirus biology. Our findings based on synthetic genomic EBOV ssRNA mimic suggests that both pathways are likely activated by filovirus RNA and MDA5 pathway is even more sensitively inhibited by VP24 than the RIG-I pathway. This may be explained by the higher signaling activity of the RIG-I pathway. It is of interest that EBOV VP35 has been shown to inhibit both RIG-I and MDA5 mediated type I IFN gene expression (40), an observation that is now extended to cover also EBOV VP24.

In summary, we have confirmed and extended previous observations on IFN antagonistic functions of filovirus VP24 proteins. We demonstrated that synthetic filovirus RNA that mimics the genomic EBOV ssRNA activates both RIG-I and MDA5 pathways to activate type I gene expression. VP24 proteins were shown to efficiently inhibit both RIG-I and MDA5 induced signaling with the MDA5-dependent pathway to be even more sensitively inhibited compared to that of the RIG-I pathway. Our observations provide baseline data for future filovirus studies and further understanding in filovirus evolution, pathogenesis and interaction with innate immunity that can be taken into account in designing novel antiviral drugs or new modalities of treatment of filovirus infections.

## Data Availability Statement

The raw data supporting the conclusions of this article will be made available by the authors, without undue reservation.

## Author Contributions

FH, MH, LK and IJ designed the study, analyzed the data and wrote the manuscript. FH, MH and HK conducted most of the transfection experiments and luciferase assays. PK designed the gene constructs and did the phylogenetic analyses. KM designed and conducted importin binding experiments and MH did transfection experiments and visualized VP24 by immunofluorescence. MQ cloned VP24 genes and verified their expression and MJ designed, produced and purified in vitro synthesized viral RNAs. SM helped with many of the experiments. All authors edited and approved the manuscript.

## Funding

This study was supported by funding from the Medical Research Council of the Academy of Finland (project numbers 297329 and 336410), Jane and Aatos Erkko Foundation (project numbers 3067-84b53 and 5360-cc2fc), Finnish Cultural Foundation, and the Sigrid Juselius Foundation.

## Conflict of Interest

The authors declare that the research was conducted in the absence of any commercial or financial relationships that could be construed as a potential conflict of interest.

## Publisher’s Note

All claims expressed in this article are solely those of the authors and do not necessarily represent those of their affiliated organizations, or those of the publisher, the editors and the reviewers. Any product that may be evaluated in this article, or claim that may be made by its manufacturer, is not guaranteed or endorsed by the publisher.

## Supplementary Material

Supplementary Figure 1 Schematic representation of the construction of EBOV ssRNA mimic. Synthetic dsDNA molecule encoding the trailer sequences 18220-18958, GFP and leader sequences 1-469 in reverse orientation was synthesized by Geneart and provided in a company-based plasmid vector pMKRQ. As described in detail in Materials and Methods the template for ssRNA synthesis was obtained by PCR followed by transcription by T7 RNA polymerase and production of EBOV genomic mimigenome ssRNA mimic that has the exact 3’ and 5’ ends of the viral genome. T7 in PCR product refers to T7 promoter.

Supplementary Figure 2 Importin α5 binding elements of Zaire ebolavirus VP24 are shared among other filovirus VP24 amino acid sequences. Identical amino acid residues within all nine filovirus VP24 proteins in the alignment are marked in red. The binding elements are marked as clusters 1, 2 and 3 based on the specific interaction between importin α5 and Zaire ebolavirus VP24 (Reference 29). Ebolavirus family VP24 proteins and Cuevavirus (Lloviu) VP24 proteins show high sequence identity in the potential importin α5 binding elements (boxed).
