# Supplementary figures and images for: Filovirus VP24 Proteins Differentially Regulate RIG-I and MDA5-Dependent Type I and III Interferon Promoter Activation
Source: Front Immunol. 2022 Jan 5;12:694105. doi: 10.3389/fimmu.2021.694105 (PMC8767557; doi:10.3389/fimmu.2021.694105)

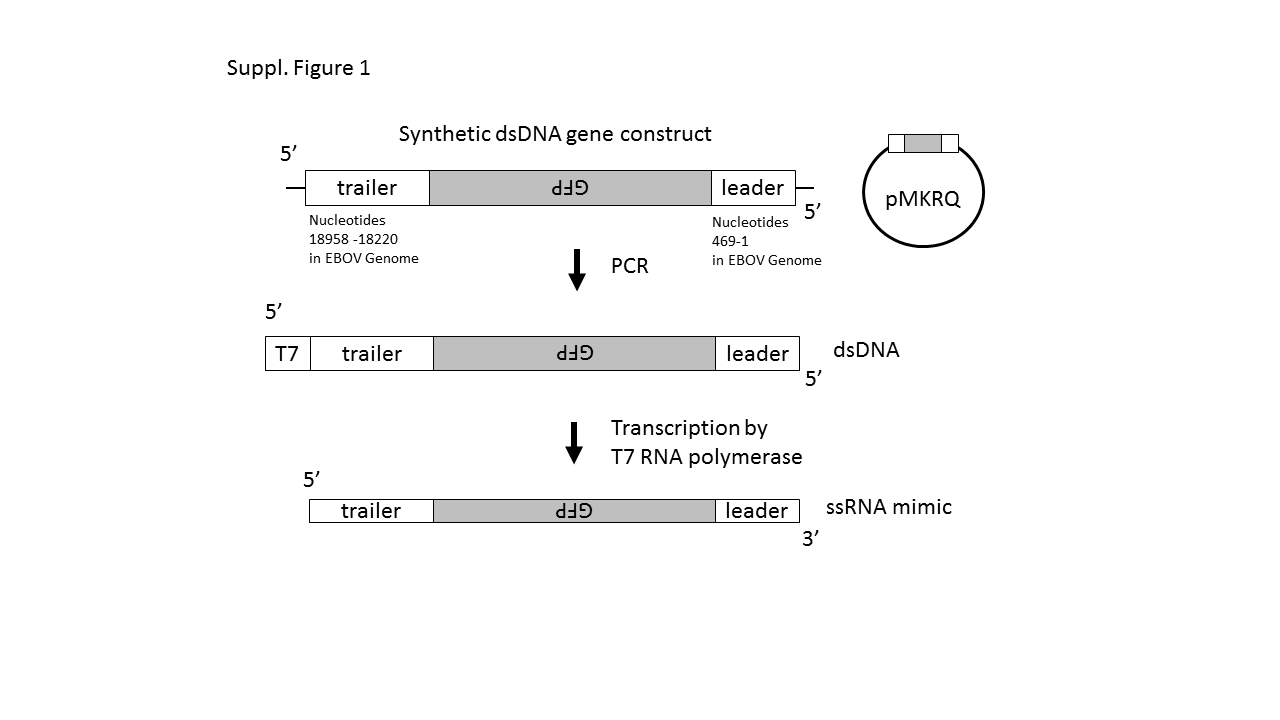

Supplement: Supplementary Figure 1 — Schematic representation of the construction of EBOV ssRNA mimic. Synthetic dsDNA molecule encoding the trailer sequences 18220-18958, GFP and leader sequences 1-469 in reverse orientation was synthesized by Geneart and provided in a company-based plasmid vector pMKRQ. As described in detail in Materials and Methods the template for ssRNA synthesis was obtained by PCR followed by transcription by T7 RNA polymerase and production of EBOV genomic mimigenome ssRNA mimic that has the exact 3’ and 5’ ends of the viral genome. T7 in PCR product refers to T7 promoter. [file Image_1.tif]

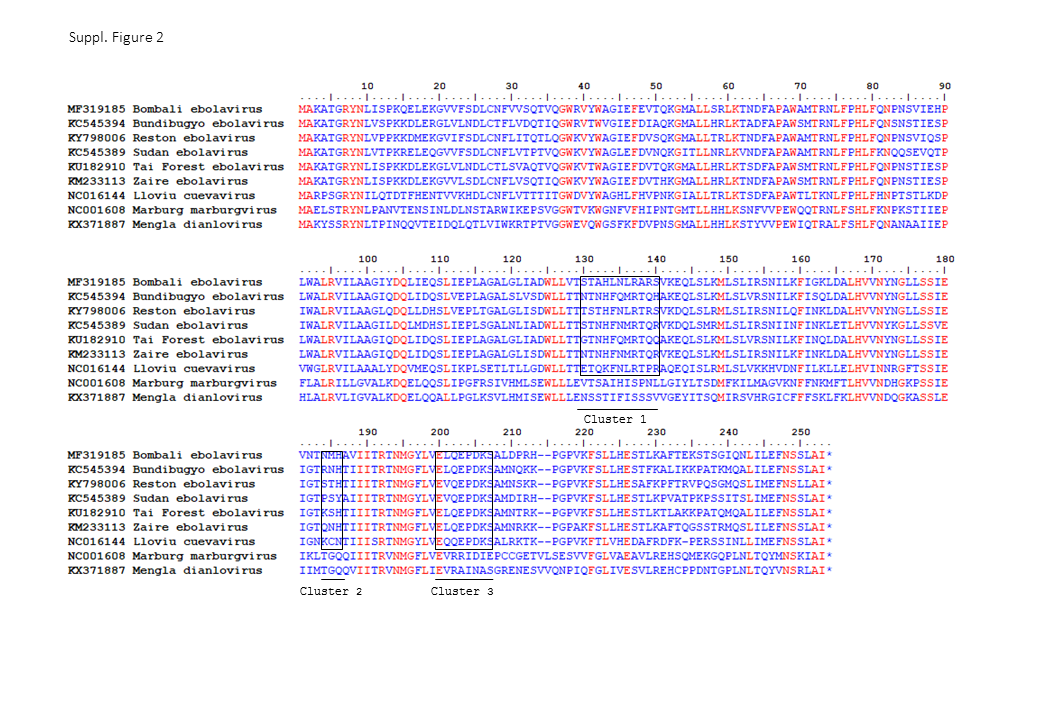

Supplement: Supplementary Figure 2 — Importin α5 binding elements of Zaire ebolavirus VP24 are shared among other filovirus VP24 amino acid sequences. Identical amino acid residues within all nine filovirus VP24 proteins in the alignment are marked in red. The binding elements are marked as clusters 1, 2 and 3 based on the specific interaction between importin α5 and Zaire ebolavirus VP24 (Reference 29). Ebolavirus family VP24 proteins and Cuevavirus (Lloviu) VP24 proteins show high sequence identity in the potential importin α5 binding elements (boxed). [file Image_2.tif]
